# The Relationship Between Sleep Quality and Shooting Accuracy in Female Archers: A Cross-Sectional Study

**DOI:** 10.3390/life16101719

**Published:** 2026-10-14

**Authors:** Aisuluu Daiyrova, Bilal Demirhan, Dzhiparkul Abdyrakhmanova, Ayça Genç, Zulfiyya Yolchiyeva Asim, Alpamys Rakhymzhanov, Aygül Çağlayan Tunç

**Affiliations:** 1 Faculty of Sports Sciences, Kyrgyz-Turkish Manas University, Bishkek 720038, Kyrgyzstan; daiyrovablossom@gmail.com (A.D.); bilal.demirhan@manas.edu.kg (B.D.); jiparkul.abdyrahmanova@manas.edu.kg (D.A.); 2 Faculty of Sports Sciences, Bartin University, 74100 Bartın, Türkiye; 3 Faculty of Physical Education, Azerbaijan Sports Academy, Baku AZ 1072, Azerbaijan; zulfiyya.yolchuyeva@sport.edu.az; 4 Sports Faculty, Khoja Akhmet Yassawi International Kazakh-Turkish University, 161200 Turkistan, Kazakhstan; alpamys.rakhymzhanov@ayu.edu.kz; 5 Faculty of Sports Sciences, İstanbul Aydin University, 34290 İstanbul, Türkiye; aygulcaglayantunc@aydin.edu.tr

**Keywords:** sleep quality, archery, shooting accuracy, precision sports, performance

## Abstract

Precision sports such as archery require sustained attention, fine motor control, and technical consistency, all of which may be influenced by sleep quality. This cross-sectional study aimed to examine the association between sleep quality and shooting accuracy in female archers and to determine whether this association persisted after adjustment for age and sport experience. Twenty-six female archers (mean age: 21.23 ± 2.35 years) participated in the study. Sleep quality was assessed using the Pittsburgh Sleep Quality Index (PSQI), and shooting accuracy (%) was calculated by normalizing the total score according to the number of shots performed. Associations between PSQI total score and shooting accuracy were examined using Pearson and Spearman correlation analyses and linear regression models. The mean PSQI total score was 6.15 ± 1.95, and the mean shooting accuracy was 77.69 ± 13.26%. Neither Pearson (*r* = 0.168, *p* = 0.413, BCa 95% CI [−0.222, 0.505]) nor Spearman correlation (ρ = 0.043, *p* = 0.835, BCa 95% CI [−0.343, 0.426]) showed a statistically significant association between PSQI total score and shooting accuracy. The adjusted regression model including age and sport experience was also not statistically significant (*R*^2^ = 0.032, *F*(3, 22) = 0.24, *p* = 0.866), and PSQI total score was not significantly associated with shooting accuracy after adjustment (B = 1.11, β = 0.163, *p* = 0.447, 95% CI [−1.86, 4.07]). In this sample of female archers, no statistically significant association was detected between habitual sleep quality assessed by the PSQI and shooting accuracy. Given the small sample size and the wide confidence intervals, larger prospective studies incorporating objective and session-specific sleep measures are needed to clarify the relationship between sleep quality and precision performance in archery.

## 1. Introduction

Sport performance is a multidimensional construct that emerges from the interaction of various physiological, biomechanical, psychological, and environmental factors. Contemporary research in sport science suggests that performance cannot be explained solely by physical components such as training load, strength, or endurance; rather, factors including sleep, recovery, stress levels, and daily lifestyle habits also play a crucial role in the sustainability and consistency of performance [1,2]. In this context, sleep quality has increasingly become a focus of research as a key lifestyle factor that can influence both the physical and cognitive performance of athletes.

Sleep is a fundamental biological requirement for the restoration of the central nervous system, regulation of cognitive functions, maintenance of hormonal balance, and neuromuscular recovery processes. Insufficient or poor-quality sleep can negatively affect several performance-related functions, including attention, reaction time, decision-making, emotional regulation, and fine motor control [3,4]. Research involving athletes suggests that poor sleep quality is a common issue, with a recent study reporting that 65% of combat sport athletes were classified as poor sleepers based on the PSQI [5]. In athletes, impairments in sleep quality have been associated with increased perceived fatigue and greater variability in performance outcomes [1]. Therefore, sleep quality is considered not only an indicator of general health, but also a key factor supporting performance stability. Previous research among college students has also demonstrated a significant association between sleep quality and psychological resilience, with poorer sleep quality being associated with lower psychological resilience [6].

A substantial body of research examining the relationship between sleep and athletic performance has primarily focused on a range of sports and performance outcomes. These studies have reported that sleep loss may negatively affect exercise performance, reaction time, perceptual performance, and cognitive functions [2], while sleep extension has been associated with improvements in sprint performance, shooting accuracy, reaction time, and mood in collegiate basketball players [7]. However, the relationship between sleep and athletic performance is not always consistent; disrupted precompetitive sleep has been associated with unfavorable mood states without a significant relationship with subsequent relative athletic performance [8].

Archery is a sport that requires a high level of technical consistency, postural stability, fine motor control, and sustained attention. The shooting process involves a complex sequence of movements, including stance, draw, aiming, release, and follow-through. Previous studies have shown that successful shooting performance is associated with specific neurophysiological and attentional processes involved in aiming and shooting [9,10].

Archery performance is commonly evaluated using the total score obtained from the arrows shot. However, raw total scores may not be directly comparable when the number of scored arrows differs. In sport science, normalising performance as a percentage of the maximum possible score is used to facilitate comparisons between participants who have completed a different number of trials [11]. Accordingly, in the present study, shooting performance was expressed as a normalized percentage of the maximum possible score to facilitate comparison across participants with different numbers of scored arrows.

A review of the literature on the relationship between sleep quality and archery performance indicates that empirical studies in this area remain limited. Most existing research has focused on the effects of sleep quality on attention, reaction time, and cognitive performance, and it has been suggested that these cognitive processes may be linked to performance in technical sports [3,12].

However, studies directly examining the relationship between habitual subjective sleep quality and normalized shooting accuracy in archers remain scarce. In particular, the association between habitual subjective sleep quality and shooting accuracy in female archers has received limited empirical attention.

In sports where technical precision is paramount, performance depends substantially on technical learning, motor control, and individual skill level. In this context, sleep quality may play a role not as a direct determinant of performance, but as a factor that supports performance stability and cognitive readiness [1,8]. Therefore, examining the relationship between sleep quality and performance using measures that reflect the technical dimension is of particular importance.

This study aimed to examine the association between sleep quality and shooting accuracy, a technical indicator of performance, in actively practicing female archers. Sleep quality was assessed using the Pittsburgh Sleep Quality Index (PSQI), and performance was evaluated based on shooting accuracy (%), calculated by normalizing total scores according to the number of shots. This approach helps reduce potential bias arising from differences in shooting volume and allows for a more consistent and comparable assessment of technical performance.

In addition to examining the unadjusted association between sleep quality and shooting accuracy, the study also aimed to determine whether this association remained after adjustment for age and sport experience. It was hypothesized that better sleep quality would be associated with higher shooting accuracy, while the strength of this association might be attenuated after adjustment for age and sport experience.

## 2. Materials and Methods

### 2.1. Participants

In this study, a cross-sectional design was used to investigate the association between sleep quality and shooting accuracy without manipulating any study variables.

Twenty-six volunteer female archers (mean age: 21.23 ± 2.35 years) participated in the study. Participants were recruited on a voluntary basis from licensed competitive archers affiliated with university teams and local archery clubs. The accessible population consisted predominantly of female archers, and only female athletes met the inclusion criteria during the data-collection period; therefore, the present study focused exclusively on female archers. The demographic characteristics of the participants are presented in Table 1.

The study was carried out in accordance with the ethical principles of the Declaration of Helsinki. The research protocol was approved by the Ethics Committee of the Kyrgyz State Academy of Physical Culture and Sport (Protocol No: 2026-23/4). Written informed consent was obtained from all participants.

Participants were eligible for inclusion if they met the following criteria:

- voluntary participation;
- having at least one year’s experience in archery;
- regular attendance at training sessions.

Participants who failed to meet one or more of the inclusion criteria listed above were excluded from the study.

### 2.2. Measured and Observed Parameters

#### 2.2.1. Sleep Quality

Sleep quality was assessed using the Pittsburgh Sleep Quality Index (PSQI), originally developed by Buysse et al. [13]. The Russian-language version of the PSQI by Semenova and Danilenko [14] was administered in the present study, consistent with its use in previous research [15]. The PSQI is a self-report instrument that assesses habitual sleep quality over the preceding month. The questionnaire was administered at the beginning of the performance-monitoring week, before any shooting-performance data were collected. The global PSQI score was calculated according to the original scoring procedure. Based on the cutoff proposed for the original PSQI, participants were categorized as having good sleep quality (PSQI ≤ 5) or poor sleep quality (PSQI > 5) [13].

#### 2.2.2. Archery Performance

In order to standardise the testing conditions, all participants shot with a recurve bow at a standard target from a distance of 18 metres under indoor competition conditions. To minimise environmental variables, all measurements were carried out indoors under constant lighting and temperature conditions (approximately 22–24 °C).

Performance data were obtained based on the total number of scored shots performed during four training sessions within a one-week period. The total number of scored shots across the four sessions ranged from 150 to 243. This approach was preferred to reduce the variability associated with single-session measurements and to provide a more representative assessment of performance.

Each session began with a warm-up consisting of blank shooting (12–18 arrows) performed at a short distance (3–5 m) without a target to prepare the muscles. This was followed by scoring/simulation shooting conducted in a format similar to competition conditions. Participants shot in ends of six arrows, with scores recorded after each end. Participants followed their regular rest intervals between ends.

Performance was quantified using shooting accuracy (%), calculated by dividing the total score by the maximum possible score based on the number of scored shots performed. To account for differences in shooting volume among participants, a normalization procedure was applied using the following formula:

Accuracy (%) = [Total Score/(Number of Scored Shots × 10)] × 100

All scored shots were evaluated according to official archery scoring rules, and scoring was recorded under the supervision of experienced coaches to ensure consistency and reliability. In addition, the use of standardized training conditions helped to minimize measurement variability.

### 2.3. Data Availability Statement

The datasets created and analysed as part of this study are not publicly available due to institutional ethical restrictions; however, they may be obtained from the corresponding author upon reasonable request.

### 2.4. Statistical Analysis

Data were analyzed using IBM SPSS Statistics version 27. Descriptive statistics were presented as mean ± standard deviation (mean ± SD), together with minimum and maximum values. Categorical variables were summarized as frequencies and percentages, and median values were also reported for the primary outcome variables. The distributional characteristics of the variables were assessed using skewness and kurtosis values, the Shapiro–Wilk test, and graphical inspection.

Participants were classified into good (PSQI ≤ 5) and poor (PSQI > 5) sleep quality groups based on their total PSQI scores. The associations between total PSQI score and shooting accuracy were examined using both Pearson’s and Spearman’s correlation analyses. In addition, a simple linear regression model and a multiple linear regression model adjusted for age and sport experience were performed. Effect estimates were reported with 95% confidence intervals (CIs). Bias-corrected and accelerated (BCa) bootstrap 95% CIs based on 5000 resamples were calculated for the correlation coefficients and adjusted regression coefficients.

All 26 participants were included in the primary analyses. Separate sensitivity analyses were conducted to assess the robustness of the findings by excluding five records in which the reported sleep duration exceeded the calculated time in bed and by alternatively scoring exactly 6 h of sleep as 2 rather than 1 in the PSQI scoring procedure. The inconsistency in these five records likely reflects the self-report and retrospective nature of the PSQI, in which participants may have misestimated their bedtime or wake-up time when completing the questionnaire. No additional clarification was requested from participants. All statistical tests were two-tailed, and statistical significance was set at *p* < 0.05.

In a sensitivity power analysis based on the Fisher z-approximation for *N* = 26 and a two-tailed α = 0.05, the smallest correlation coefficient that could be detected with 80 per cent power was calculated to be approximately |r| = 0.526.

Prior to regression analysis, the assumptions of linearity, homoscedasticity, and independence of residuals were assessed through visual inspection of residual and scatter plots; no obvious violations were detected. Multicollinearity was considered unlikely given the small number of predictors and their conceptual independence.

### 2.5. Generative AI Statement

During the preparation of this article, generative artificial intelligence was used solely for the purposes of language editing, grammar correction and text abridgement. Generative artificial intelligence was not used in the study design, data collection, data analysis, data interpretation or the creation of any tables or results.

## 3. Results

A total of 26 female archers were included in the primary analyses, with no missing data for any of the variables analyzed. Descriptive statistics for participant characteristics, sleep-related variables, and shooting performance are presented in Table 1. The mean age of the participants was 21.23 years (standard deviation [SD] = 2.35; range = 18–25 years), and the mean sport experience was 4.27 years (SD = 1.19; range = 3–7 years).

The mean PSQI total score was 6.15 (*SD* = 1.95; 95% confidence interval [CI] = 5.36–6.94; *Mdn* = 6.00), with scores ranging from 2 to 9. Based on the PSQI > 5 cutoff, 10 participants (38.5%) were classified as having good sleep quality, whereas 16 (61.5%) were classified as having poor sleep quality. The mean shooting accuracy was 77.69%, with values ranging from 43.20% to 94.46%.

Correlations between PSQI total score and shooting accuracy are presented in Table 2. The Pearson correlation coefficient indicated a weak positive correlation, which was not statistically significant (*r* = 0.168, *p* = 0.413, BCa 95% CI [−0.222, 0.505]). Similarly, the Spearman correlation showed no statistically significant association (ρ = 0.043, *p* = 0.835, BCa 95% CI [−0.343, 0.426]).

The results of the simple linear regression (Model 1) and the multiple linear regression adjusted for age and sport experience (Model 2), examining the association between PSQI total score and shooting accuracy, are presented in Table 3. Model 1 was not statistically significant (*R*^2^ = 0.028, *F*(1, 24) = 0.69, *p* = 0.413). In this model, a one-point increase in PSQI total score was associated with a 1.14-percentage-point increase in shooting accuracy; however, the coefficient was not statistically significant and its confidence interval included zero (B = 1.14, β = 0.168, *p* = 0.413, 95% CI [−1.68, 3.96]).

After adjustment for age and sport experience, Model 2 was also not statistically significant (*R*^2^ = 0.032, *F*(3, 22) = 0.24, *p* = 0.866). In Model 2, PSQI total score (B = 1.11, β = 0.163, *p* = 0.447, 95% CI [−1.86, 4.07], BCa 95% CI [−1.47, 3.46]), age (B = 0.83, β = 0.147, *p* = 0.773), and sport experience (B = −1.41, β = −0.126, *p* = 0.804) were not significantly associated with shooting accuracy. The PSQI total score coefficient changed only slightly after adjustment for age and sport experience (from 1.14 to 1.11). The adjusted *R*^2^ values were negative for both models (Model 1: −0.012; Model 2: −0.100), indicating that, after accounting for the number of predictors, neither model provided additional explanatory value compared with an intercept-only model.

Sensitivity analyses were conducted to assess the robustness of the findings. Excluding the participant with the lowest shooting accuracy (43.20%; n = 25), the association between PSQI total score and shooting accuracy remained nonsignificant (r = 0.188, *p* = 0.369; adjusted B = 0.992, 95% CI [−1.505, 3.488], *p* = 0.418).

## 4. Discussion

The present study examined the association between subjective sleep quality and shooting accuracy in female archers. No statistically significant association was observed between PSQI total score and shooting accuracy in either the Pearson or Spearman correlation analyses (Table 2). Similarly, PSQI total score was not significantly associated with shooting accuracy in the unadjusted model or after adjustment for age and sport experience (Table 3). However, the confidence intervals were relatively wide and included both negative and positive values. Therefore, the present findings should not be interpreted as evidence that no relationship exists between sleep quality and shooting accuracy.

The present findings should be considered within the broader literature on sleep and athletic performance. Previous research has indicated that insufficient or poor-quality sleep may adversely affect physical and cognitive performance, injury risk, and recovery [16]. A recent systematic review and meta-analysis also reported that acute sleep deprivation can negatively affect several domains of athletic performance, although the magnitude of these effects may vary according to the type of performance outcome, timing of sleep loss, and characteristics of the athletes studied [17]. These findings provide a general rationale for investigating sleep in precision sports such as archery, where successful performance depends not only on physical capacity, but also on sustained attention, motor control, and technical consistency.

Evidence specifically concerning archery remains limited. Guru et al. [18] examined sleep patterns and sports performance in adolescent archers using a pre–post-intervention design and reported improvements in sleep-related parameters and aspects of archery performance following the intervention. More recently, Prasetyo et al. [19] investigated sleep extension and precision performance in young archers under field conditions. Although longer sleep was accompanied by a descriptive improvement in precision performance, the phase-related change in performance was not statistically supported. Thus, the limited archery-specific literature does not provide entirely consistent evidence regarding the magnitude of the relationship between sleep and shooting performance.

One possible explanation for the difference between the present findings and some previous studies is the nature of the sleep exposure being examined. Previous intervention studies have examined performance responses to experimentally altered or extended sleep over different intervention periods [17,18,19]. In contrast, the PSQI assesses habitual subjective sleep quality over the preceding month. Consequently, the absence of a statistically significant association in the present study may indicate that habitual subjective sleep quality does not correspond directly to the acute sleep state experienced immediately before shooting. This distinction is particularly relevant in precision sports, in which performance on a given day may be more sensitive to recent or session-specific sleep than to a broader retrospective assessment of habitual sleep.

Studies in other precision- and skill-dependent sports also suggest that the relationship between sleep and accuracy may depend on the type and timing of sleep disturbance. Edwards and Waterhouse [20] reported that partial sleep deprivation affected the accuracy and consistency of dart throwing, while Reyner and Horne [21] demonstrated that sleep restriction impaired serving accuracy in tennis players. These findings suggest that acute sleep loss can influence accuracy-based performance. However, they are not directly comparable with the present study because they examined experimentally induced or short-term sleep restriction rather than habitual subjective sleep quality.

The broader sports literature likewise shows that findings are not entirely consistent. Cunha et al. [22], in a systematic review of sleep interventions and athletic performance, identified sleep extension and napping as among the more promising approaches, while some studies of napping and other sleep-related interventions reported no significant performance effects. Similarly, another systematic review and meta-analysis [23] found improvements in subjective sleep quality and reductions in sleepiness following sleep interventions, whereas significant improvements were not consistently observed for objective sleep measures or aerobic and anaerobic performance outcomes. A systematic review focusing specifically on sleep extension also concluded that potential performance benefits varied according to the performance outcome examined and emphasized limitations in the quality of the available evidence [24]. Taken together, some studies indicate that sleep restriction may impair performance or that extending sleep may be beneficial [17,18,20,21,22,24], whereas other findings show no statistically significant effects for certain sleep interventions or only nonsignificant trends in performance [19,22,23]. Therefore, the available evidence indicates variability in the magnitude and consistency of sleep-related effects on athletic performance, while evidence specifically concerning precision- and skill-dependent outcomes remains limited.

Differences in sleep assessment methods may further contribute to variation across studies. Sleep in athletes has been assessed using a wide range of subjective and objective approaches, including questionnaires, sleep diaries, actigraphy, and polysomnography [25,26]. Simim et al. [25] highlighted considerable methodological variability in the monitoring and definition of sleep quality among individual-sport athletes, while Vlahoyiannis et al. [26] emphasized the methodological considerations and limitations associated with different approaches to sleep assessment in athletic populations. In the present study, sleep quality was assessed using the PSQI; therefore, the results primarily reflect habitual subjective perceptions of sleep rather than objectively measured sleep immediately preceding shooting performance.

The temporal relationship between the sleep and performance measurements should also be considered. The PSQI was administered at the beginning of the performance-monitoring week and refers to sleep during the preceding month, whereas shooting performance was subsequently monitored over one week. Accordingly, the sleep measure and the performance measure did not represent exactly the same temporal period. This temporal mismatch may have attenuated an association if shooting accuracy was influenced more strongly by sleep during the night immediately preceding a shooting session or by short-term fluctuations in sleep duration and quality.

From an applied perspective, coaches and practitioners should consider that an athlete’s habitual sleep quality may not reflect their sleep state on the night before competition. Acute sleep monitoring (e.g., sleep diaries or actigraphy on the night before competition) may therefore provide more relevant information for performance preparation than retrospective assessments of habitual sleep. This distinction is particularly important in precision sports such as archery, where performance on a given day may be more sensitive to recent sleep than to broader sleep patterns.

From a physiological and motor-control perspective, archery performance requires precise coordination and stable control of the bow arm. Ertan [27] demonstrated distinct muscular activation patterns of the bow-hand forearm during recurve archery shooting, with differences observed between elite and beginner archers. In addition, sleep deprivation has been reported to interfere with mechanisms involved in postural regulation and balance [28]. These observations provide a plausible basis for expecting sleep-related changes to influence precision performance. Nevertheless, the present study did not directly measure postural control, muscle activation, or other neuromuscular variables; therefore, these mechanisms remain possible explanations rather than mechanisms demonstrated by the present data.

Neurocognitive mechanisms may also be relevant. Sleep deprivation has been associated with alterations in attention, vigilance, working memory, and executive functioning [29,30], functions that may contribute to maintaining concentration and technical consistency during repeated shooting. However, acute sleep deprivation and habitual subjective sleep quality represent different sleep constructs. Thus, evidence demonstrating neurocognitive impairment following experimentally induced sleep loss cannot be assumed to translate directly into a linear association between PSQI scores and shooting accuracy under habitual training conditions.

The adjusted regression analysis provides an additional perspective on these findings. After age and sport experience were included in the model, PSQI total score remained nonsignificantly associated with shooting accuracy (Table 3). However, the model explained only a small proportion of the variance in shooting accuracy. Archery performance is multifactorial, and variables not included in the present analysis—such as technical skill, training load, competitive level, psychological state, fatigue, chronotype, menstrual-cycle-related factors, and sleep immediately preceding the shooting sessions—may have contributed to individual differences in performance. The nonsignificant association observed in the present study may therefore partly reflect unmeasured variability rather than the absence of a sleep–performance relationship.

Sensitivity analyses also supported the robustness of the primary statistical result. Excluding participants whose reported sleep duration exceeded time in bed and applying an alternative scoring approach for sleep duration did not materially alter the direction or statistical significance of the findings. Nevertheless, these analyses address specific scoring and data-consistency issues and do not overcome the broader limitations associated with sample size, subjective sleep assessment, and the cross-sectional design.

### Limitations

Certain limitations should be borne in mind when interpreting the current findings. Firstly, the relatively small sample size (*N* = 26) has resulted in wide confidence intervals, which has limited the precision with which the direction and magnitude of the relationship between sleep quality and shooting accuracy can be estimated. As indicated by the sensitivity power analysis, the study was sufficiently powered only for large associations; therefore, the statistically nonsignificant findings should not be interpreted as evidence that no relationship exists. Secondly, the study included only female archers, and performance was assessed in a standardised 18-metre indoor recurve setting; this limits the generalisability of the findings to male athletes, other archery disciplines, distances and the wider archery population. Furthermore, the participants’ competitive level and technical skills were not systematically measured. Thirdly, sleep was assessed using the PSQI, a subjective and retrospective measurement tool; objective measurements such as actigraphy or polysomnography were not available. Fourthly, the PSQI assessment period did not coincide with the one-week shooting performance monitoring period. Fifthly, potentially relevant factors such as training load, fatigue, chronotype, psychological state, menstrual-cycle-related factors (which were not assessed in the present study), caffeine intake and sleep immediately prior to individual shooting sessions were not systematically controlled for. One participant had a notably lower shooting accuracy (43.20%) than the rest of the sample; however, this value was retained in the primary analyses to preserve the full variability of the sample. Sensitivity analysis excluding this participant did not materially change the findings. Finally, due to the cross-sectional design, the findings reflect associations rather than causality.

## 5. Conclusions

In conclusion, no statistically significant association was detected between subjective sleep quality and shooting accuracy among the female archers included in this study. Similarly, sleep quality was not significantly associated with shooting accuracy after adjustment for age and sport experience. However, the wide confidence intervals indicate uncertainty regarding the direction and magnitude of the association; therefore, these findings should not be interpreted as evidence of the absence of a relationship. Larger prospective studies incorporating objective and session-specific sleep measures are needed to clarify the relationship between sleep quality and shooting performance in archers.

## Figures and Tables

**Table 1:** Descriptive statistics for participant characteristics, sleep variables and shooting performance (*N* = 26).

Variable	*M*	*SD*	Min	Max
*Participant characteristics*				
Age (years)	21.23	2.35	18	25
Sport experience (years)	4.27	1.19	3	7
Weight (kg)	63.96	9.95	49	89
Height (cm)	168.54	7.02	150	180
BMI (kg/m^2^)	22.49	2.95	17.71	29.40
*Sleep variables*				
Sleep latency (min)	23.46	18.15	5	60
Sleep duration (h)	6.83	0.87	6.00	9.00
Time in bed (h)	7.18	1.16	4.50	9.00
PSQI total score	6.15	1.95	2	9
*PSQI component scores*				
C1 Subjective sleep quality	1.50	1.07	0	3
C2 Sleep latency	1.04	1.08	0	3
C3 Sleep duration	0.77	0.43	0	1
C4 Habitual sleep efficiency	0.23	0.43	0	1
C5 Sleep disturbances	1.12	0.52	0	3
C6 Use of sleeping medication	0.12	0.43	0	2
C7 Daytime dysfunction	1.38	0.94	0	3
*Shooting performance*				
Number of shots	220.31	27.06	150	243
Total score	1716.42	361.85	648	2267
Shooting accuracy (%)	77.69	13.26	43.20	94.46

*M* = mean; *SD* = standard deviation; BMI = body mass index; PSQI = Pittsburgh Sleep Quality Index. PSQI component scores range from 0 to 3, and the total score ranges from 0 to 21, with higher scores indicating poorer sleep quality. Time in bed was calculated from reported bedtime and wake-up time, while shooting accuracy (%) was calculated using the following formula: Total score/(Number of shots × 10) × 100.

**Table 2:** Pearson and Spearman correlations between PSQI total score and shooting accuracy.

Coefficient	Estimate	*p*	BCa 95% CI
Pearson *r*	0.168	0.413	[−0.222, 0.505]
Spearman ρ	0.043	0.835	[−0.343, 0.426]

CI = confidence interval; BCa = bias-corrected and accelerated. Bootstrap estimates were based on 5000 resamples. *p*-values are two-tailed. Higher PSQI total scores indicate poorer sleep quality, whereas higher shooting accuracy indicates better performance; therefore, a positive coefficient indicates that higher PSQI total scores are associated with higher shooting accuracy values.

**Table 3:** Linear regression models predicting shooting accuracy (*N* = 26).

Predictor	*B*	*SE*	*β*	*p*	95% CI for *B*	BCa 95% CI for *B*
Model 1: R^2^ = 0.028, adjusted R^2^ = −0.012, F(1, 24) = 0.69, *p* = 0.413						
Constant	70.69	8.81	—	<0.001	[52.52, 88.87]	—
PSQI total score	1.14	1.37	0.168	0.413	[−1.68, 3.96]	—
Model 2: R^2^ = 0.032, adjusted R^2^ = −0.100, F(3, 22) = 0.24, *p* = 0.866						
Constant	59.32	40.04	—	0.153	[−23.72, 142.36]	[−27.70, 138.94]
PSQI total score	1.11	1.43	0.163	0.447	[−1.86, 4.07]	[−1.47, 3.46]
Age	0.83	2.83	0.147	0.773	[−5.05, 6.70]	[−6.52, 9.00]
Sport experience	−1.41	5.62	−0.126	0.804	[−13.07, 10.24]	[−12.12, 8.08]

Dependent variable: Shooting accuracy (%). *B* = unstandardized coefficient; *SE* = standard error; β = standardized coefficient; CI = confidence interval; BCa = bias-corrected and accelerated bootstrap (5,000 resamples). BCa confidence intervals were calculated only for Model 2. *p*-values are two-tailed.

## Data Availability

The data presented in this study are available upon request from the corresponding author due to ethical restrictions.

## Abbreviations

The following abbreviations are used in this manuscript:

PSQI	Pittsburgh Sleep Quality Index
BMI	Body Mass Index
BCa	Bias-Corrected and Accelerated
CI	Confidence Interval
